# Supplementary material for: Image reconstruction through a multimode fiber with a simple neural network architecture
Source: Sci Rep. 2021 Jan 13;11:896. doi: 10.1038/s41598-020-79646-8 (PMC7806887; doi:10.1038/s41598-020-79646-8)
Supplement: Supplementary file 1 — Supplementary information [file 41598_2020_79646_MOESM1_ESM.pdf]

Optical Society of America under the terms of the OSA Open Access Publishing Agreement

Supplementary Materials for:

Image reconstruction through a multimode fiber with a simple neural network architecture

Changyan Zhu, Eng Aik Chan, You Wang, Weina Peng, Ruixiang Guo, Baile Zhang, Cesare Soci, and Yidong Chong.

1. SSIM and MSE

The SSIM (Structural Similarity Index) is a commonly-used perceptual metric based on visible structures in the image, developed by Wang *et al.* [?]. It is defined as

$$\mathrm{SSIM}(x,y) = l(x,y)^{\alpha} \cdot c(x,y)^{\beta} \cdot (x,y)^{\gamma} \tag{S1}$$

where

$$l(x,y)^{\alpha} = \frac{2\mu_x\mu_y + c_1}{\mu_x^2 + \mu_y^2 + c_1}, \quad c(x,y) = \frac{2\sigma_x\sigma_y + c_2}{\sigma_x^2 + \sigma_y^2 + c_2}, \quad s(x,y) = \frac{\sigma_{xy} + c_3}{\sigma_x\sigma_y + c_3} \tag{S2}$$

are measures for luminance, contrast, and structural similarity respectively. Here, x and y denote two images to be compared, μ_x and μ_y are their average values, σ_x and σ_y are the standard deviation, and σ_{xy} is the covariance. We set $c_1 = (k_1 L)^2$, $c_2 = (k_2 L)^2$, and $c_3 = c_2/2$. $k_1 = 0.01$ and $k_2 = 0.03$. $L = 1$ is the dynamic range of the pixel-values. α, β, and γ are the weights for each feature, which we set to 1.

When training the neural networks (NNs), we use the SSIM to define a loss function according to

$$Loss_{\mathrm{SSIM}} = 1 - \mathrm{SSIM}. \tag{S3}$$

Another measure of the similarity between two images X and Y, the Mean Squared Error (MSE), is defined as

$$MSE = \frac{1}{MN}\sum_{1}^{M}\sum_{1}^{N}[X(m,n) - Y(m,n)]^2 \tag{S4}$$

As shown in Fig. 3 of the main text, the SSIM and MSE produce similar results when used to evaluate NN performance.

2. Neural network settings

To optimize the performance of each NN, we tested several different hyperparameter choices.

2.1. SHL-DNN versus multilayer perceptron

In several applications of dense neural networks (DNNs), it is advantageous to increase the depth of the NN since this increases the representational capacity of the network. In the context of multimode fiber (MMF) image reconstruction, we investigated the performance of dense networks with different depths.

Fig. S1(a) shows the training curves for two dense NNs with different numbers of hidden layers, but the same total number of trainable parameters. The SHL-DNN has

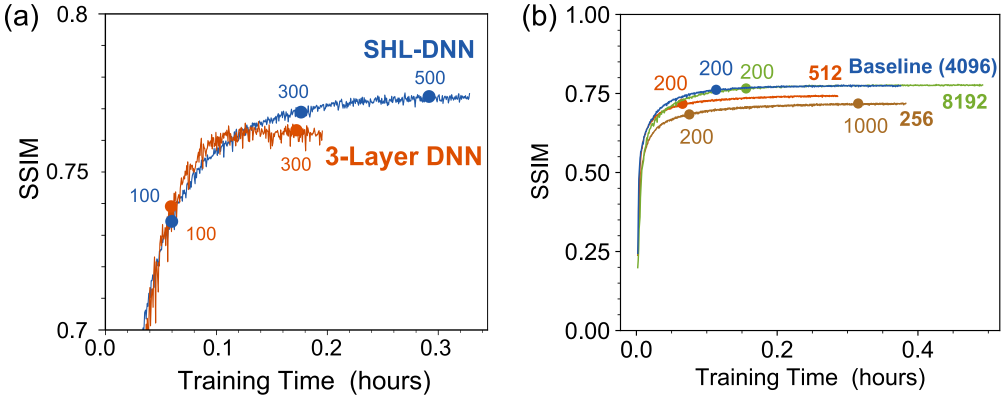

Fig. S1. (a) Training curves for dense neural networks with a single hidden layer (blue) and 3 hidden layers (orange). The networks have the same total number of trainable parameters. (b) Training curves for SHL-DNNs with different numbers of hidden nodes: 256 (brown), 512 (orange), 4096 (baseline, blue), and 8192 (green).

4096 nodes in the hidden layer, and the 3-layer DNN contains 2164 nodes per hidden layer. We use the same early-stopping condition (100 epochs after validation losses stop improving). The SHL-DNN saturates at SSIM of 0.775, significantly higher than the 3-layer DNN, which has SSIM of 0.766. Hence, a multilayer structure does not appear to be advantageous for image reconstruction fidelity.

2.2. *Number of hidden layer nodes in SHL-DNN*

In Fig. S1(b), we plot the training curves SHLs with different numbers of hidden layer nodes (256, 512, 4096, and 8192). It can be seen that negligible improvement is achieved by going from 4096 to 8192 nodes.

2.3. *Activation units in SHL-DNN*

Fig. S2(a) shows the training curves for SHL-DNNs with different activation units in the hidden layer: tanh, ReLU, and sigmoid. The sigmoid activation appears to perform the best, and this was the one we used for the SHL-DNNs in the main text.

Also shown here is the training curve for a SHL-DNN without a sigmoid activation in the output layer – i.e., sigmoid activation only in the hidden layer (labelled "single sigmoid", and plotted in green). This also leads to worse performance than the baseline configuration.

2.4. *Choice of objective function*

The NNs in the main text are trained by maximizing the SSIM as the objective function. We also investigated other choices of objective function, including MSE loss and cross entropy loss. These do not appear to offer any significant advantage for either type of NN. As an example, Fig. S2(b) shows the training curves (evaluated in terms of SSIM loss) for SHL-DNNs with different choices of objective function.

2.5. *U-net filter number optimization*

In optimizing the U-Net, the performance noticeably improves as the number of filters is increased up to "4×", (i.e., 4× the number of filters used in Ref. [**?**]), as shown in Fig. 3(e)

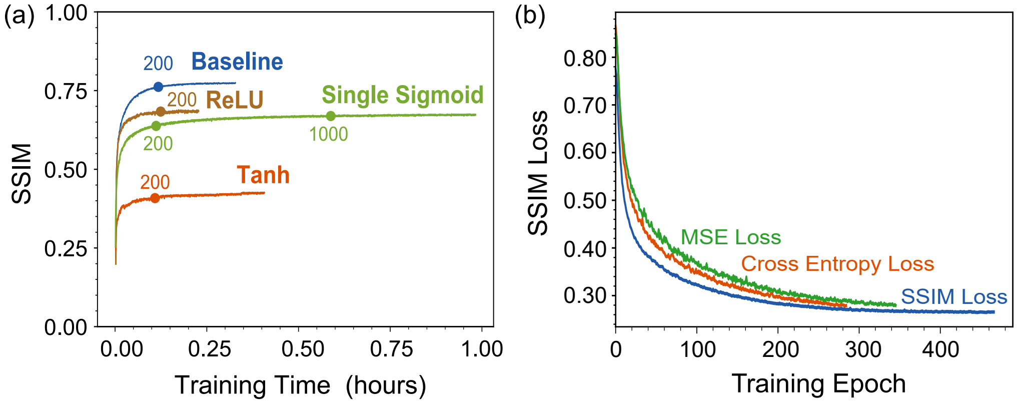

Fig. S2. (a) Training curves for SHL-DNNs with different kinds of activation units: double sigmoid (baseline, blue), ReLU (brown), tanh (orange), and sigmoid only in the hidden layer (green). (b) Training curves for SHL-DNNs optimized using different loss functions, evaluated in terms of SSIM loss: SSIM loss (blue), cross entropy (orange), and MSE (green).

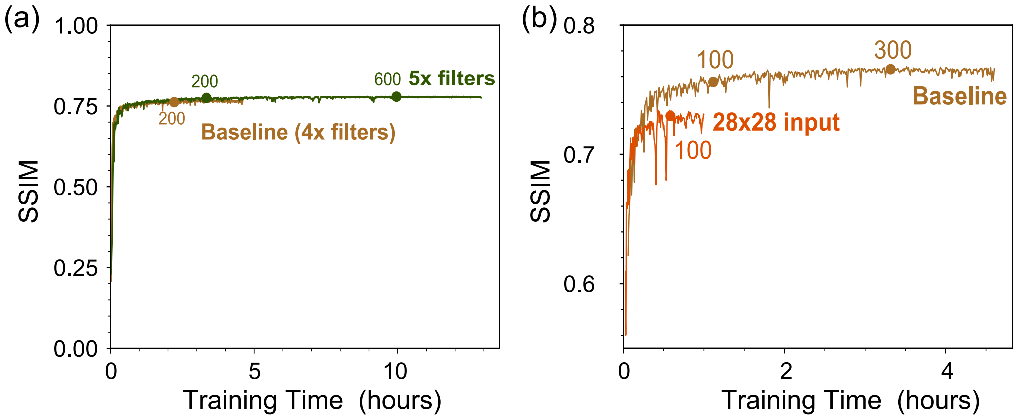

Fig. S3. (a) Training curve for U-Net with 4× and 5× the number of filters (relative Ref. [?]). The U-Net with 4× filters is the baseline configuration featured in the main text, and achieves similar performance to the U-Net with 5× filters with significantly less training time. (b) Training curves for U-Net with 64×64 inputs (baseline, brown) and with 28×28 inputs (orange). In both cases, the ground truth and output images are 28×28.

of the main text. Upon increasing the filter number from 4× (31 million trainable parameters) to 5× (49 million trainable parameters), the saturated SSIM exhibits a very small improvement while the training time increases substantially, as shown in Fig. S3(a). Therefore, we use 4× filters in our baseline U-Net configuration.

2.6. U-Net with symmetric input-output

The U-Net described in the main text is "asymmetric", in the sense that the input and output images have different sizes. It is reasonable for the output images to have the same size as the ground truth images (28×28), but there is no good reason to limit the input (speckle) images to that same size; they can be downscaled from the resolution of the camera image to any desired size. In the main text, we took the input images to be

64×64.

We also investigated the performance of a "symmetric" U-Net, in which both the inputs, outputs, and ground truth images are all 28×28. This smaller input size necessitated removing a pair of contracting and expanding convolutional blocks (the third and fifth convolutional blocks in Fig. 1(d) in the main text). We use the same early-stopping criterion (i.e., stopping 100 epochs after the validation curve stops improving). As shown in Fig. S3(b), the resulting U-Net performs significantly worse, with SSIM 0.734 compared to SSIM 0.767 for the baseline U-Net (with 64x64 inputs).

3. Performance of SHL-DNN and U-Net on Fashion Mnist dataset

In Fig. 3 and 4 of the main text, we compared the SHL-DNN and U-Net performance for the MNIST digits dataset. In Fig. S4(c), we show the same results for the MNIST-Fashion dataset. Similar to the previous conclusions, the U-Net does not outperform the SHL-DNN in reconstructed image fidelity (as measured by the SSIM), while taking much longer to train.

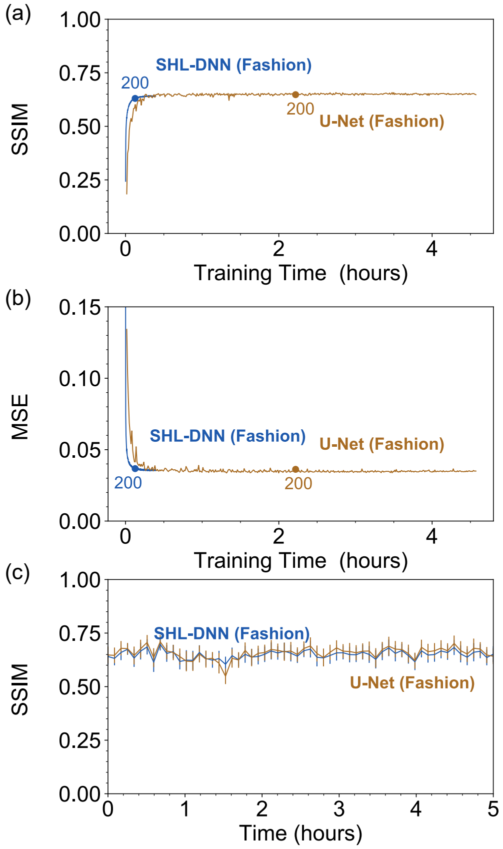

Fig. S4. Comparison of SHL-DNN (blue) and U-Net (brown) for the Fashion MNIST data set. (a) SSIM versus training time. (b) MSE versus training time (c) SSIM of reconstructed images using data collected up to 5 hours subsequent to the training set.
